# Study on the effect of rubber content on the frost resistance of steel fiber reinforced rubber concrete

**DOI:** 10.1038/s41598-024-64719-9

**Published:** 2024-06-15

**Authors:** Lei Jiang, Jiahua Jing, Ming Zhang, Shuai Yang

**Affiliations:** 1 College of Civil Engineering and Architecture, Anyang Normal University, Anyang, 455000 Henan China; 2 Engineering Technology Research Center of Henan Province for Digital Intelligent Building and Low Carbon Building Material, Anyang, 455000 Henan China

**Keywords:** Steel fiber reinforced rubber concrete, Freeze–thaw cycles, Damage layer thickness, Pore structure, Civil engineering, Composites

## Abstract

In cold areas, the steel fiber reinforced rubber concrete (SFRRC) pavement is exposed to natural environment and experiences varying degrees of damage from freezing and thawing. This can have a serious impact on the normal usage and safe operation of the pavement structure. This research examines the impact of varying rubber concentrations on multiple variables, such as the rate of mass reduction, relative dynamic modulus of elasticity, compressive strength, and thickness of the damage layer (*H*_f_) during freeze–thaw (F-T) durability testing conducted on SFRRC. Furthermore, an analysis is conducted to determine the degradation pattern exhibited by SFRRC. The internal structure evolution and pore structure characteristics of SFRRC were examined using scanning electron microscopy and mercury intrusion porosimetry techniques, which revealed the underlying damage mechanism in SFRRC during F-T cycles. The results suggest that the addition of an appropriate amount of rubber can effectively enhance the frost resistance of SFRRC in water. A gradual improvement in the frost resistance of SFRRC is observed when increasing the rubber content from 0 to 10%. The optimal frost resistance is observed in SFRRC with 10% rubber content. However, when the rubber content reaches 15%, SFRRC exhibits significant degradation and lower level of resistance to freezing compared to SFRRC without rubber. Microcracks form within SFRRC due to the freezing–thawing forces experienced during the experiment, resulting in the development of a damage layer that extends from the surface to the interior. The compressive strength of the damaged layer significantly decreases as *H*_f_ increases. The addition of appropriate rubber in SFRRC improves its pore structure, leading to an increased proportion of harmless or less harmful pores and a reduction in average pore size, thereby significantly enhancing its frost resistance.

The insolubility of rubber materials in water and organic substances, combined with their slow decomposition rate, leads to significant environmental pollution and substantial waste of resources. Rubberized concrete, which involves incorporating waste rubber particles or powder into the concrete mixture, is a type of elastic concrete that exhibits excellent impact resistance, damping capabilities, and fatigue performance. This material has already found practical applications in engineering^1–4^. However, experimental studies and practical engineering experience have shown that the incorporation of rubber in concrete leads to a reduction in compressive strength, splitting tensile strength, and flexural strength. These adverse effects have implications for the widespread adoption of rubber concrete^5–8^.

To address this, both domestic and international studies have examined methods for incorporating high-modulus-of-elasticity steel fibers into rubber concrete as reinforcement material in order to mitigate the reduction in mechanical strength caused by the presence of rubber. The steel fiber reinforced rubber concretes have demonstrated promising results. The findings from Zhao et al.^9^ suggest that increasing the dosage of steel fibers significantly improves both the compressive strength and elastic modulus of rubberized concrete. Additionally, Peng et al.^10^ also demonstrate that an increase in the volume fraction of steel fibers effectively enhances not only the compressive strength and elastic modulus but also the splitting tensile strength and flexural strength within a range between 2.7% to 37.1%. Alsaif et al.^11^ have demonstrated that the incorporation of steel fibers into rubberized concrete effectively minimizes the reduction in flexural strength caused by the addition of rubber, decreasing it from 50% to only 9.6% compared to ordinary concrete. Yan et al.^12^ indicated that the inclusion of steel fibers within rubberized concrete exhibits a significant supplementary impact on its compressive strength, achieving a maximum enhancement of approximately 25.1%.

Currently, most of the research on SFRRC focuses on its mechanical properties. However, durability is an important indicator for determining whether an engineering material can be widely and effectively applied. With the increasing utilization of SFRRC application, neglecting its durability will lead to premature deterioration in structural performance and a reduction in service life. Professor Mehta^13^ has pointed out that the degradation of concrete structures can mainly be attributed to several factors, including the corrosion of steel reinforcement, damage caused by F-T action in cold climates, and erosive environmental interactions involving both physical and chemical processes.

One of the main application areas of SFRRC is pavement concrete structures, which are exposed to the natural environment for a long time and a large area. Consequently, they are highly susceptible to the influence of atmospheric precipitation and temperature fluctuations. According to statistical data, approximately 53.5% of areas in China experience a climate characterized by seasonal freezing^14^. In regions with cold weather conditions, the pavement constructed using SFRRC materials may suffer varying degrees of damage caused by F-T action, which significantly impacts the regular usage and safe operation of the pavement structure.

In recent years, researchers have conducted investigations into the challenges posed by F-T issues in concrete reinforced with steel fibers or rubber. Niu et al.^15,16^ suggested that incorporating steel fibers into concrete could enhance its resistance to spalling and mitigate the rate of strength reduction following freezing–thawing cycles. According to Miao et al.^17^, it has been demonstrated that steel fiber reinforced concrete exhibits superior freezing resistance compared to conventional concrete; however, its enhancement effect is less pronounced in comparison to air-entraining agent. Both the size and content of rubber particles play crucial roles in determining the durability of rubberized concrete. While incorporating rubber particles into the mixture may result in a decrease in strength, it can enhance resistance to freezing. Richardson et al.^18^ emphasized the beneficial role of rubber particles smaller than 0.5 mm in enhancing air entrainment and increasing the freezing resistance of rubber concrete. Gesoğlu et al.^19^ conducted a study on the freezing resistance of rubber concrete, examining the influence of different rubber contents (10% and 20%). The findings indicated that reducing the percentage of rubber in the mixture significantly improves its ability to withstand freezing conditions. According to Gonen^20^, the size of rubber particles has a significant influence on the F-T resistance of concrete. The incorporation of fine rubber powder has a notable positive impact on enhancing the F-T resistance of concrete, similar to the effects observed when using air-entraining agents. Zhang et al.^21^ conducted a study on the pore structure and found that an increase in rubber content significantly enhances the air-entraining effect of rubber particles.

There is limited research on the F-T durability of SFRRC in comparison to rubber concrete and steel fiber reinforced concrete. The study conducted by Wang et al.^22^ suggests that the incorporation of rubber fibers and steel fibers into self-compacting concrete can greatly enhance its resistance to F-T cycles, even up to 600 cycles, while effectively controlling the expansion of specimens during this process. Li^23^ examined the influence of steel fibers on the mechanical and impermeability properties of SFRRC and discovered that adding steel fibers can significantly enhance both the splitting tensile strength and flexural strength of rubberized concrete. Specifically, when the volume fraction of steel fibers reaches 1.5%, it achieves optimal impermeability performance. Wang^24^ investigated the influence of different proportions of steel fiber and rubber powder on the resistance of SFRRC to F-T cycles. The findings revealed that as the content of steel fiber and rubber powder increased, the mass loss and relative dynamic modulus loss over 200 F-T cycles decreased more gradually, resulting in improved F-T resistance. Concrete incorporating 1.5% steel fiber and 15% rubber powder demonstrated better F-T performance. Li^25^ investigated the influence of rubber particle size and content on the F-T behavior of steel fiber secondary graded concrete in a sodium chloride solution. The results indicated that, after 48 cycles of salt F-T exposure and within the range of rubber content from 0 to 20%, the chloride F-T resistance of rubber-modified steel fiber secondary graded concrete with smaller-sized rubber particle sizes (1–3 mm) increased with the increase in rubber content. Notably, this resistance exceeded that of rubber-modified steel fiber secondary graded concrete with larger-sized rubber particles (3–6 mm).

It is evident that there has been limited research on the microstructural characteristics and durability of SFRRC when exposed to F-T conditions. In response to the practical need for waste rubber in pavement engineering and the environmental importance of recycling resources, as well as further enriching the research data on durability of SFRRC in cold regions, this study systematically conducted experimental research on the frost resistance performance of SFRRC with different rubber content in water. Compared to the existing literature, this study utilized rubber particles measuring 1–2 mm in size as fine aggregate. By considering *H*_f_ as an evaluation index for SFRRC damage and combining it with mass, RDME, and compressive strength, the degradation patterns of SFRRC were discussed from a macro physical performance perspective. The effects of F-T cycles on the structural changes and pore characteristics of SFRRC are also investigated using scanning electron microscopy (SEM) and mercury intrusion porosimetry (MIP) techniques. Furthermore, it reveals the underlying mechanisms responsible for the damage inflicted on SFRRC in F-T environments.

## Materials and methods

### Materials

**Figure 1:**
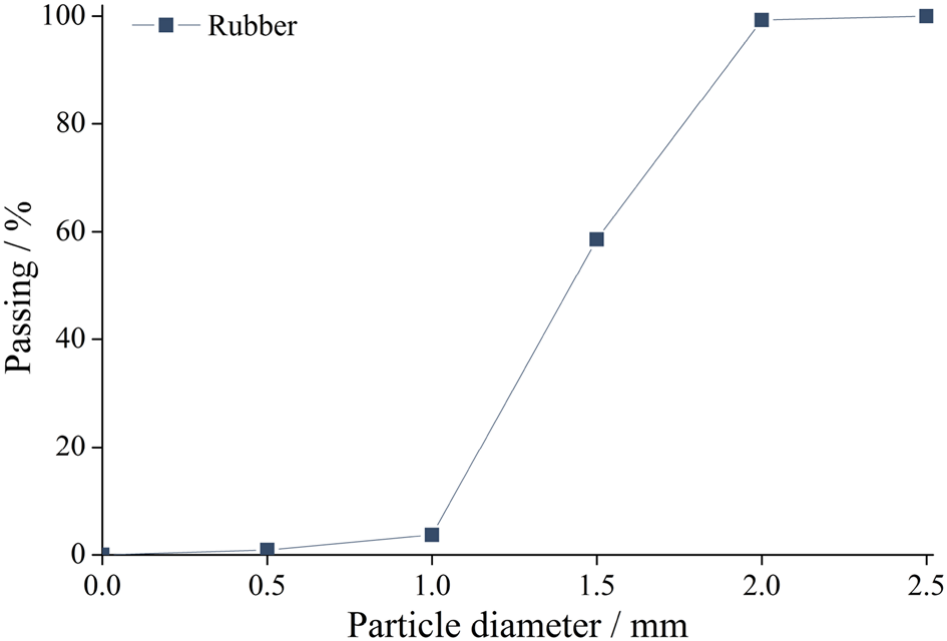
Particle size distribution of rubber.

**Figure 2:**
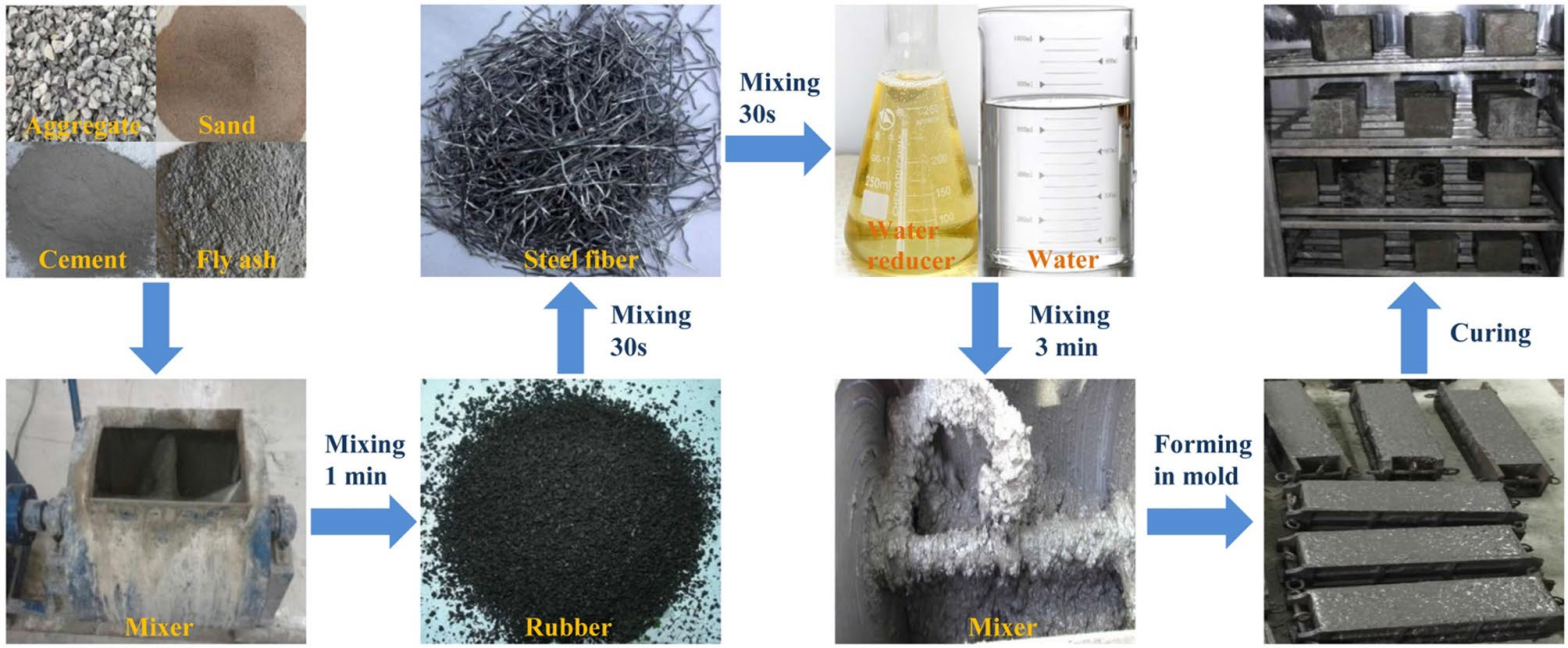
Flow chart of SFRRC specimens preparation.

The experiment utilized Portland cement with a grade of 42.5 and grade II fly ash, which met the material quality requirements specified in GB 175-200725^26^ and GB/T 1596-201726^27^, respectively. The chemical characteristics of the cement and fly ash used in this study are summarized in Table 1. The fine aggregate consisted of standard river sand with a fineness modulus of 2.7, while the coarse aggregate comprised graded gravel ranging from 5 to 20 mm in size. Rubber particles measuring 1–2 mm in diameter were added to replace a portion of the fine aggregate. These rubber particles, sourced from a rubber factory located in Tianjin, have an apparent density of 1050 kg/m^3^. The distribution of their particle sizes is depicted in Fig. 1, while the technical indicators for their physical and chemical properties are provided in Table 2. Additionally, the appearance of the rubber particles is shown in Fig. 2. To strengthen the concrete, wave-shaped steel fibers with a length-to-diameter ratio of 60 were utilized (as shown in Fig. 2). Furthermore, a polycarboxylic acid water reducer was applied to reduce water content by up to 30%. Tap water served as the mixing water source.

**Table 1:** Chemical composition of cement and fly ash.

Constituent (wt.%)	SiO_2_	Al_2_O_3_	CaO	MgO	SO_3_	Fe_2_O_3_	Na_2_O	K_2_O
Cement	19.5	4.76	64.68	2.16	3.02	3.25	0.34	1.36
Fly ash	50.72	28.78	6.93	1.21	1.26	4.23	1.33	1.97

**Table 2:** Technical indexes of rubber aggregate.

Properties (%)	Moisture content	Fiber content	Metal content	Ash content	Acetone extract	Rubber hydrocarbon content	Carbon black content
Values	0.6	0.2	0.02	6	10	52	33

### Specimen preparation

To prepare the SFRRC specimens, a water-binder ratio of 0.45 was employed. Fine aggregate was partially replaced with rubber, at replacement percentages of 0%, 5%, 10%, and 15% respectively, using equal volume-based proportions. Additionally, the mixture contained steel fibers at a volume fraction of 1.5%. Detailed mix proportions for the SFRRC utilized in the experiment can be found in Table 3.

**Table 3:** Mix proportion of concrete /(kg·m^−3^). The figures after R indicate the proportion of rubber particles replacing fine aggregate by equal volume, 0%, 5%, 10% and 15%, respectively.

Sample types	Cement	Fly ash	Sand	Coarse aggregate	Water	Steel fiber	Rubber	Water reducer
SFR0C	366	41	704	1056	183	117	0.0	4.07
SFR5C	366	41	690	1056	183	117	13.9	4.07
SFR10C	366	41	677	1056	183	117	27.2	4.07
SFR15C	366	41	664	1056	183	117	40.1	4.07

The procedure for preparing the SFRRC specimens is depicted in Fig. 2. To begin with, the cement, fly ash, sand, and coarse aggregate were placed in the mixer and mixed for a duration of 1 min. Then, rubber particles were introduced to the mixture and mixed for an additional 30 s. Immediately after that, steel fibers were evenly added and mixed for another 30 s. Finally, water containing a water-reducing agent was added and the mixture was mixed for 3 min before being poured into molds and vibrated. After a 24-h period, the specimens were removed from the molds and placed under controlled curing conditions maintained at 20 ± 3 °C with a relative humidity of 95% for a duration of 28 days. Subsequently, an additional curing phase was conducted at room temperature for another period of 60 days.

### Testing methodologies

#### Freeze–thaw test

**Figure 3:**
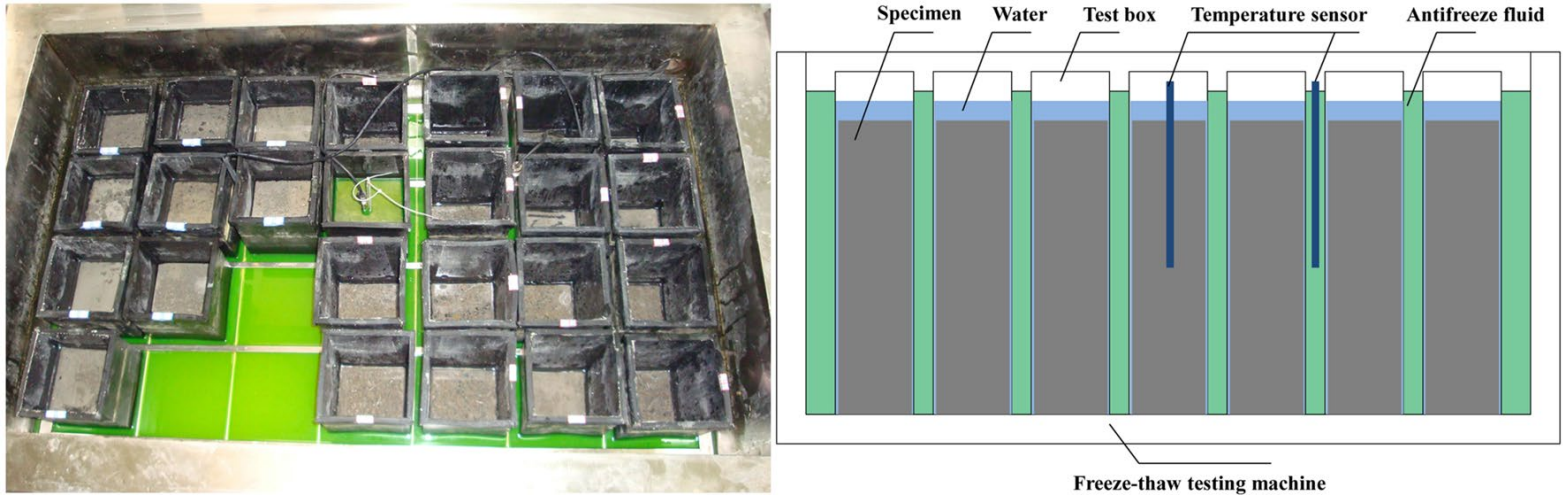
SFRRC specimens in F-T test.

The rapid F-T test, based on GB/T50082-2009^28^, was conducted to evaluate the freezing resistance of the SFRRC. After completing the curing process, the specimens were immersed in water for 4 days. They were then subjected to a rapid F-T testing machine to initiate the testing procedure, as shown in Fig. 3. Inside the F-T testing machine, there are 28 rubber specimen boxes. Each box can accommodate either four 100 mm × 100 mm × 100 mm cubic specimens or one 100 mm × 100 mm × 400 mm prism specimen. Antifreeze fluid is injected into the F-T testing machine to control the temperature through 2 temperature sensors embedded in the test specimens and antifreeze fluid. The temperature range during this test varied from − 18 ± 2 °C to 5 ± 2 °C. It is required that each cycle of freezing and thawing be completed within a timeframe of 2–4 h, with the duration for thawing being at least one-fourth of the total time for freezing and thawing.

After every 25 F-T cycles, prisms with dimensions of 100 mm × 100 mm × 400 mm were used to evaluate both the mass and the relative dynamic modulus of elasticity (RDME), while cubes measuring 100 mm × 100 mm × 100 mm were employed to determine the compressive strength. The testing procedure involved three replicate specimens, and the results were analyzed based on the average values obtained. If the RDME falls below 60% or if the rate of mass reduction exceeds 5%, or once the total number of F-T cycles reaches 300, the experiment should be stopped.

The mass of the specimen was measured using an electronic scale capable of achieving an accuracy of 0.5 g. Subsequently, the percentage of mass loss could be determined by employing the following Eq. (1).

1 $$\Delta M_{{\text{N}}} = \left[ {\frac{{\left( {m_{0} - m_{{\text{N}}} } \right)}}{{m_{0} }}} \right] \times 100\% ,$$

where *ΔM*_N_ represents the percentage decrease in the mass of the specimen (%); *m*_0_ and *m*_N_ represent the mass (kg) of the specimen before testing and after *N* cycles of F-T testing, respectively.

**Figure 4:**
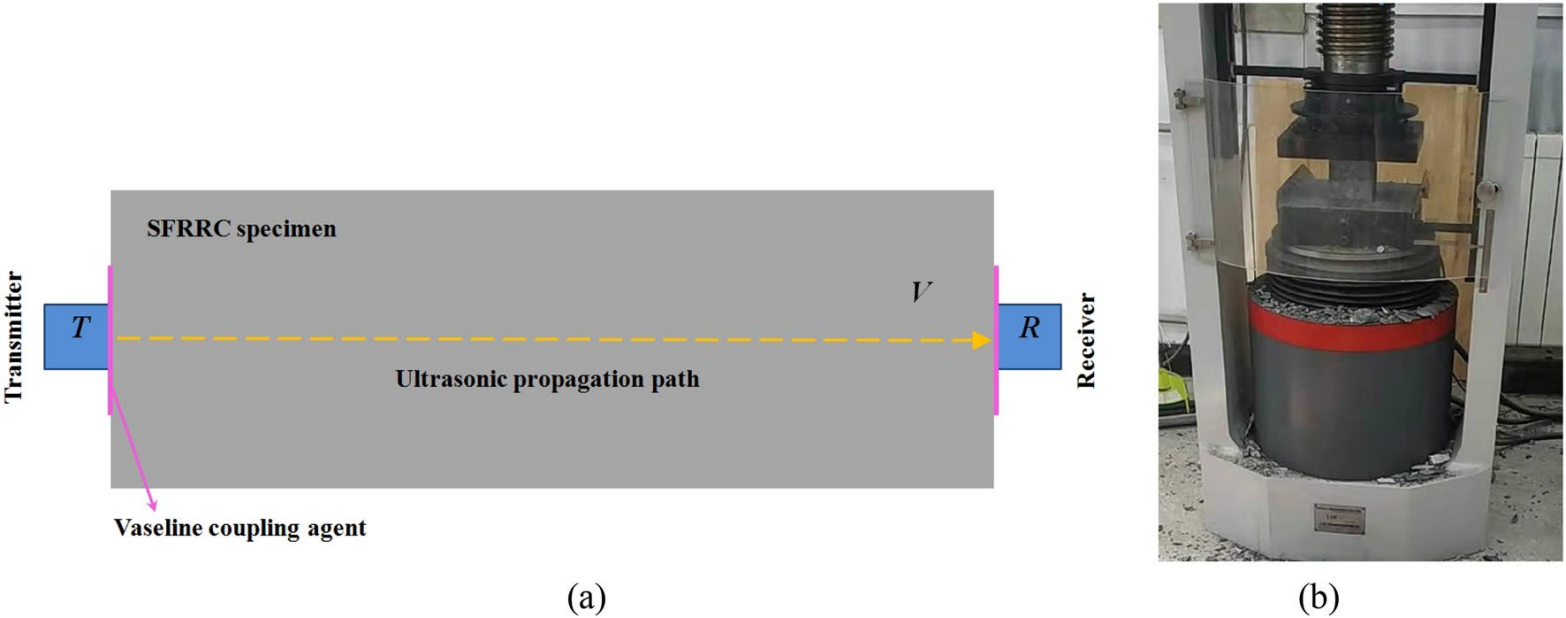
Test of RDME and compressive strength: (**a**) RDME test; (**b**) Compressive strength test.

A non-metallic ultrasonic detector was utilized to conduct RDME assessment on the specimen, employing a direct method of ultrasonic penetration as depicted in Fig. 4a. The RDME of specimen can be determined using Eq. (2)^29^.

2 $$RE_{{\text{N}}} = \frac{{E_{{\text{N}}} }}{{E_{{0}} }} = \frac{{t_{0}^{2} }}{{t_{N}^{2} }} \times 100\% ,$$

where *RE*_N_ represents the RMDE of the specimen (%); *E*_0_ and *E*_N_ represent the dynamic elastic modulus (GPa) of the specimen before testing and after *N* cycles of F-T testing, respectively; *t*_0_ and *t*_N_ represent the time (μs) taken for the ultrasonic wave to travel through the specimen before testing and after *N* cycles of F-T testing.

The digital display pressure testing machine, shown in Fig. 4b, was used to determine the compressive strength of the specimen. The loading rate ranged from 0.5 MPa/s to 0.8 MPa/s during the experiment. The relative compressive strength of the specimen can be obtained by Eq. (3).

3 $$Rf_{{\text{N}}} = f_{{\text{N}}} /f_{{0}} ,$$

where R*f*_N_ represents the relative compressive strength (%) of the specimen; *f*_0_ and *f*_N_ represent the compressive strength (MPa) of the specimen before testing and after *N* cycles of F-T testing, respectively.

#### Damage layer thickness

When concrete structures are exposed to frost damage, fire, or chemical erosion, the outer layer of concrete develops microcracks and gradually deteriorates. As a result, a damage layer forms, which negatively impacts both the structural integrity and long-term durability of the concrete constructions. As ultrasonic measurement technology has advanced, the application of ultrasonic methods for assessing the *H*_f_ of concrete has gained acceptance. In the study referenced as^30^, the utilization of both chemical analysis and ultrasonic measurement techniques was employed to investigate the *H*_f_ resulting from sulfate erosion, effectively validating the practicality of employing the ultrasonic method. Additionally, previous studies^31,32^ have examined the variations in *H*_f_ for both conventional concrete and gangue concrete under F-T conditions. The *H*_f_ in SFRRC was determined using a non-metallic ultrasonic detector, as referenced in literature^33,34^. The prism specimens measuring 100 mm × 100 mm × 400 mm were utilized for this purpose. The *H*_f_ and ultrasonic wave velocity in the damage layer were measured after 100 F-T cycles, and then every 50 cycles, until 300 cycles.

**Figure 5:**
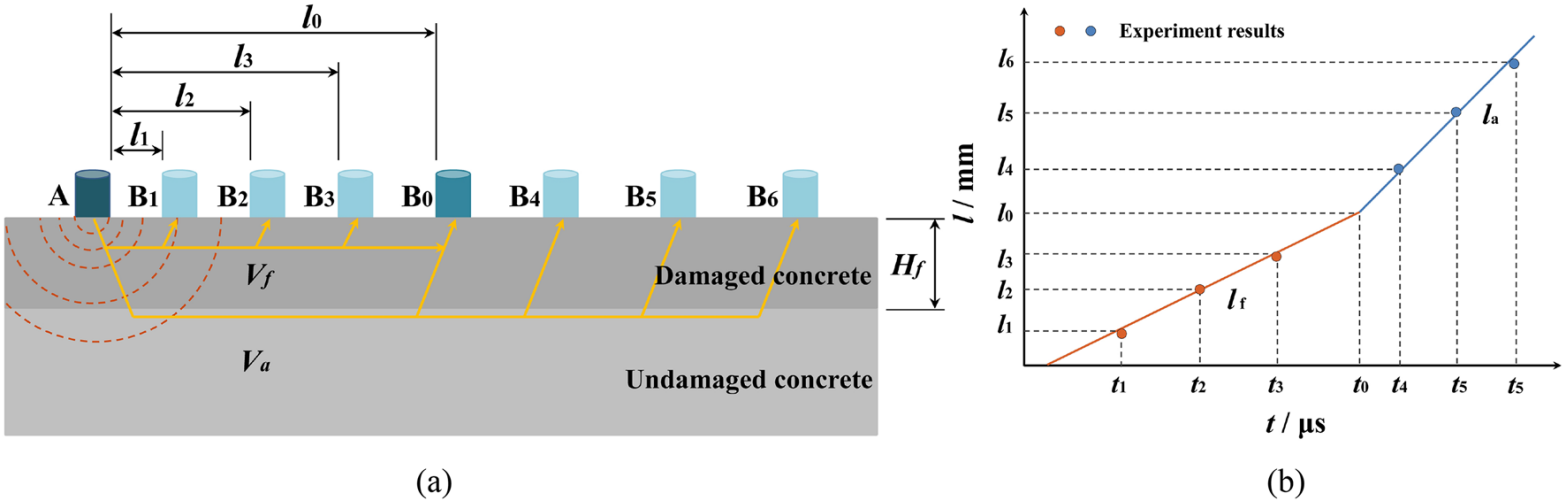
Test method for *H*_f_: (**a**) Setting of the transducers; (**b**) Relationship between propagation time and transducer distance.

As depicted in Fig. 5a, the transmitting transducer is positioned at point A, and the distance between it and the edge of the test specimen remains constant at 50 mm. The receiving transducer gradually moves towards the other end with an interval of 50 mm, passing through points such as B1, B2, B3 until it reaches a distance of 300 mm.

At a specific distance *l*_0_, the time taken for ultrasonic waves to traverse both the damaged and undamaged layers is exactly equal to the time it takes for the waves to propagate directly through only the damaged layer. Figure 5b illustrates the correlation between transducer distance and propagation time. Therefore, the *H*_f_ is determined by Eqs. (4) and (5).

4 $$H_{{\text{f}}} = \frac{{l_{0} }}{2}\sqrt {\frac{{V_{{\text{a}}} - V_{{\text{f}}} }}{{V_{{\text{a}}} + V_{{\text{f}}} }}} ,$$

5 $$l_{0} = \frac{{A_{{\text{f}}} V_{{\text{a}}} - A_{{\text{a}}} V_{{\text{f}}} }}{{V_{{\text{a}}} - V_{{\text{f}}} }},$$

where *V*_a_ and *V*_f_ respectively represent the velocities of ultrasonic waves (km/s) in the undamaged layer and damaged layer, which can be determined by examining the slopes of *l*_a_ and *l*_f_ in Fig. 5b; *A*_a_ and *A*_f_ can be determined by examing the intercepts of *l*_a_ and *l*_f_ in Fig. 5b.

**Figure 6:**
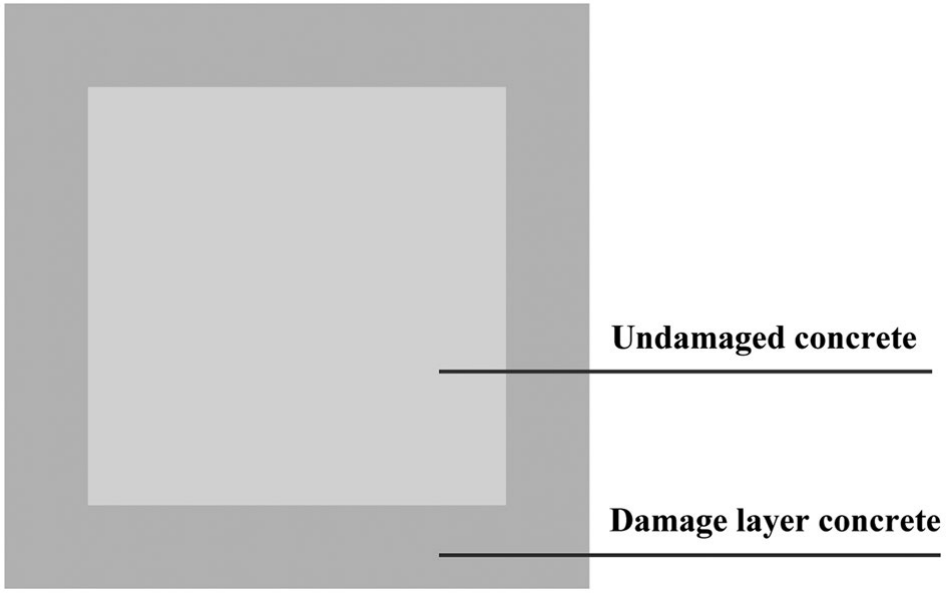
Cross-section distributions of damaged SFRRC.

SFRRC damaged by F-T cycles consists of an external damage layer and internal undamaged concrete, as shown in Fig. 6. Assuming an even distribution of these two parts, the compressive strength satisfies the following equation:

6 $$f_{{\text{c}}}^{\prime} A = f_{{\text{c}}} A_{{\text{c}}} + f_{{\text{d}}} A_{{\text{d}}}$$

where *f*_c,_ *f*_d_, and $f_{{\text{c}}}^{\prime}$ represent the compressive strengths (MPa) of the undamaged concrete, the damage layer concrete, and the concrete after F-T test (composed of damage layer and undamaged concrete), respectively; and *A*_c_, *A*_d_, and *A* are the sectional areas (mm^2^) of the undamaged concrete, the damage layer and the concrete after F-T test (composed of damage layer and undamaged concrete), respectively, where $A_{{\text{c}}} = (100 - 2H_{{\text{f}}} )^{2} {\text{mm}}^{2}$, $A_{{\text{d}}} = (A - A_{{\text{c}}} ){\text{mm}}^{2}$, *A* = 10^4^mm^2^.

#### SEM test

After completing the compressive strength test, the cement mortar paticles with an approximate size of 5 mm were selected and immersed in anhydrous ethanol to prevent further hydration. Prior to conducting the experiment, the sample was removed from the ethanol solution and dried in a vacuum oven at a temperature of 110℃ for a duration of 24 h. Subsequently, it was placed in a desiccator and cooled to room temperature. Finally, the micro-morphology of SFRRC was observed using SEM.

#### MIP analysis

The pore structure was analyzed using MIP. Firstly, the cement mortar without aggregate was selected from the specimen, with the diameter of the cement mortar particles not exceeding 5 mm. Subsequently, the sample was preserved in anhydrous ethanol to prevent further hydration and chemical reactions. Prior to testing, the sample was allowed to air-dry naturally for 2 h, followed by drying in a vacuum oven at 110 °C for 24 h. After the drying procedure, the sample was gradually cooled to room temperature inside a desiccator. Then, it was placed in a sealed bag, ready for testing.

## Results and discussion

### Change in mass loss

**Figure 7:**
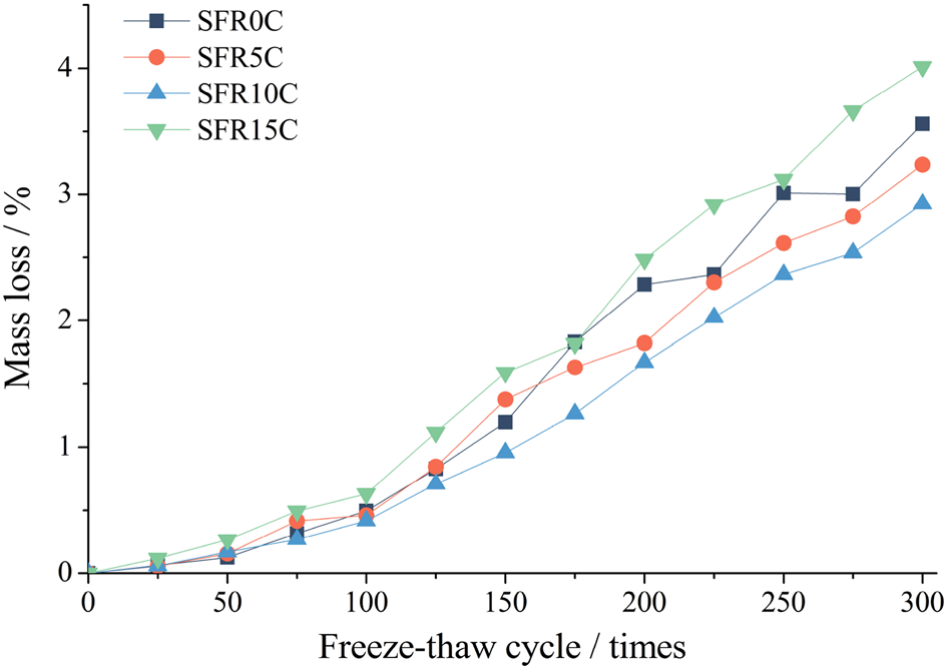
Mass loss of SFRRC.

Figure 7 illustrates the influence of varying rubber content levels on the mass reduction of SFRRC during F-T cycles. It is shown that the rate of mass loss increases slowly during the initial stages of the experiment and accelerates significantly after 100 cycles. The respective mass losses for SFR0C, SFR5C, SFR10C, and SFR15C are recorded as 3.36%, 3.24%, 2.92%, and 4.01% after 300 cycles. It is clear that there is a gradual decrease in the rate of mass loss as the rubber content increases from 0 to 10%. Notably, SFR10C exhibits the lowest mass loss and demonstrates superior resistance against F-T peeling. However, the mass loss rate significantly increases when the rubber content reaches 15%, surpassing even that of SFR0C without rubber, which exhibits the lowest F-T peeling resistance.

As an organic elastomer, rubber has a dual effect on the frost resistance of SFRRC. On the positive side, due to its high toughness, rubber possesses significant tensile and compression deformation abilities. This characteristic helps reduce hydrostatic pressure and osmotic pressure during F-T processes, effectively mitigating concrete crack expansion and reducing connectivity. Simultaneously, the inclusion of rubber particles acts as an air-entraining agent by increasing the quantity of enclosed small pores in the concrete^20^. Consequently, this reduces frost heave stress within the concrete and improves its resistance against freezing conditions. On the negative side, due to its organic nature, the chemical inertness of the rubber surface leads to insufficient adhesion between rubber and cement^22^. This can result in a weak interface layer that is prone to cracking and failure under frost heave stress. Therefore, adding an appropriate amount of rubber into concrete has been found effective in enhancing its frost resistance. However, excessive amounts of rubber tend to increase the occurrence of weak interface layers, thus reducing its frost resistance.

### Change in RDME

**Figure 8:**
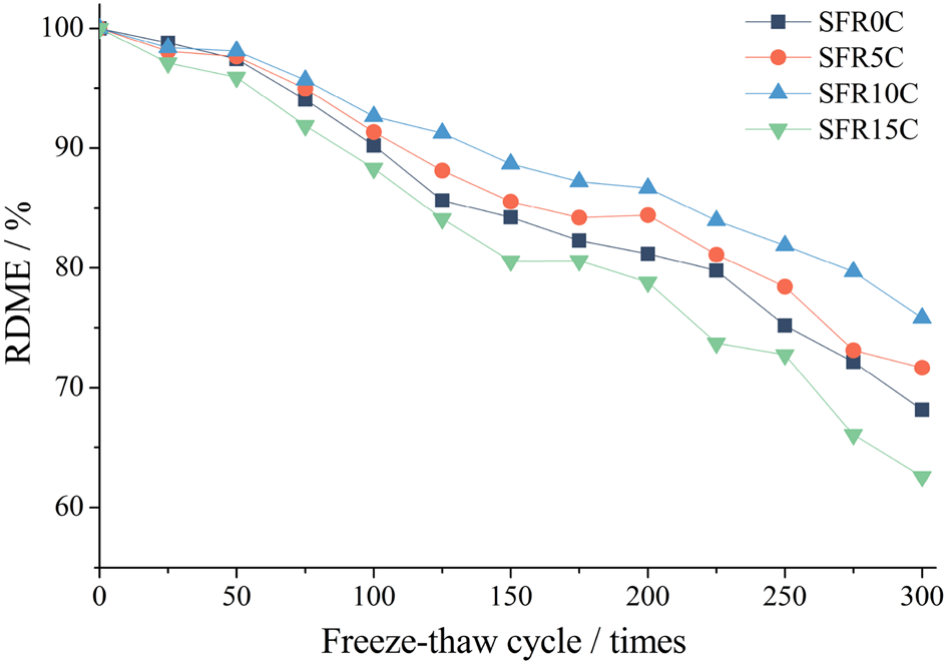
Relative dynamic modulus of elastically of SFRRC.

Figure 8 illustrates the influence of varying rubber content levels on the RDME loss of SFRRC during F-T cycles. It can be observed that during the initial phase of the test, there is a slow decrease in RDME, followed by a significant decrease in the subsequent phase.After undergoing 300 cycles, the RDMEs of SFR0C, SFR5C, SFR10C and SFR15C are reduced to 68.18%, 71.69%, 75.81% and 62.57% respectively. As the rubber content is increased from 0 to 10%, a gradual decrease in the RDME is observed. Notably, SFR10C exhibits minimal loss in RDME and demonstrates superior frost resistance performance compared to other types of SFRRC. However, increasing the rubber content to 15% results in a significant increase in RDME loss. This can be attributed to the higher rubber content, which leads to an increase in internal defects within SFR15C and consequently decreases its frost resistance performance. During the first 100 F-T cycles, the bond between steel fibers and the concrete matrix remains strong, effectively maintaining crack resistance and causing a slow decline in RDME. However, with repeated application of temperature stress and frost heave stress, the bond strength at the interface between the steel fibers and the matrix weakens, leading to a reduction in crack resistance^17^. Subsequently, as more cracks emerge and their width increases, SFRRC experiences an accelerated decrease in RDME.

The decrease in RDME primarily results from an increase in cracks and a decrease in compactness within SFRRC. Cracks serve as the main channels for water and ice crystals to expand and damage the concrete. During crack propagation, randomly distributed rubber particles exhibit a blocking effect, while staggered steel fibers help alleviate stress at crack tips, thereby inhibiting further growth of the crack. Additionally, elastic rubber and high-strength steel fibers can absorb and disperse part of the stress during F-T cycles^35^. The combined effect significantly enhances the frost resistance of SFRRC.

### Change in compressive strength

**Figure 9:**
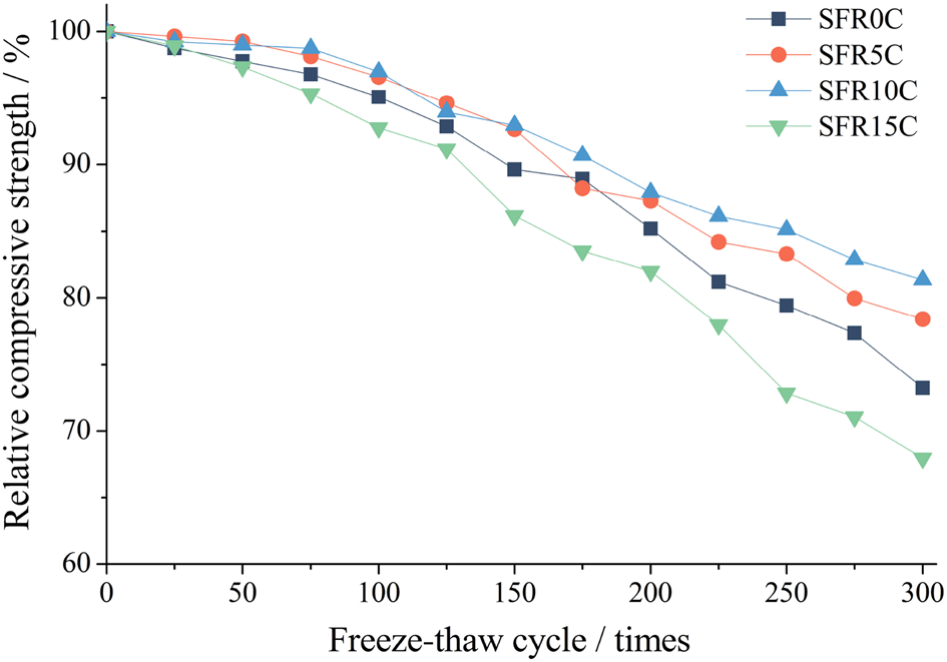
Relative compressive strength of SFRRC.

Figure 9 illustrates the influence of varying rubber content levels on the compressive strength of SFRRC during F-T cycles. After 300 cycles, the compressive strengths of SFR0C, SFR5C, SFR10C, and SFR15C decrease to 73.22%, 78.36%, 81.36%, and 67.98% respectively. As the proportion of rubber in SFRRC increases from 0 to 10%, a gradual decrease in compressive strength is observed. Among different types of specimens, SFR10C exhibits the least decrease in relative compressive strength. The incorporation of rubber particles at this level significantly enhances the internal pore structure, leading to a predominantly positive influence on SFR10C and mitigating the reduction in compressive strength.

However, incorporating 15% rubber into SFR15C results in a significant decrease in the relative compressive strength compared to SFR0C without the addition of rubber. This phenomenon can be attributed to two main reasons. Firstly, rubber particles, as elastomers, have low strength. The inclusion of rubber particles at this higher content increases weak defects within the SFR15C, thereby reducing its effective bearing area and resulting in a reduction of its mechanical properties^12^. Secondly, rubber, due to its high-molecular organic nature, exhibits a relatively weak bonding interface between its particles and cement mortar. When subjected to external forces, concrete tends to fail along this interfacial zone^8^. Therefore, a higher rubber content adversely affects the concrete, resulting in a faster decline in compressive strength.

### Change in damage layer thickness

**Figure 10:**
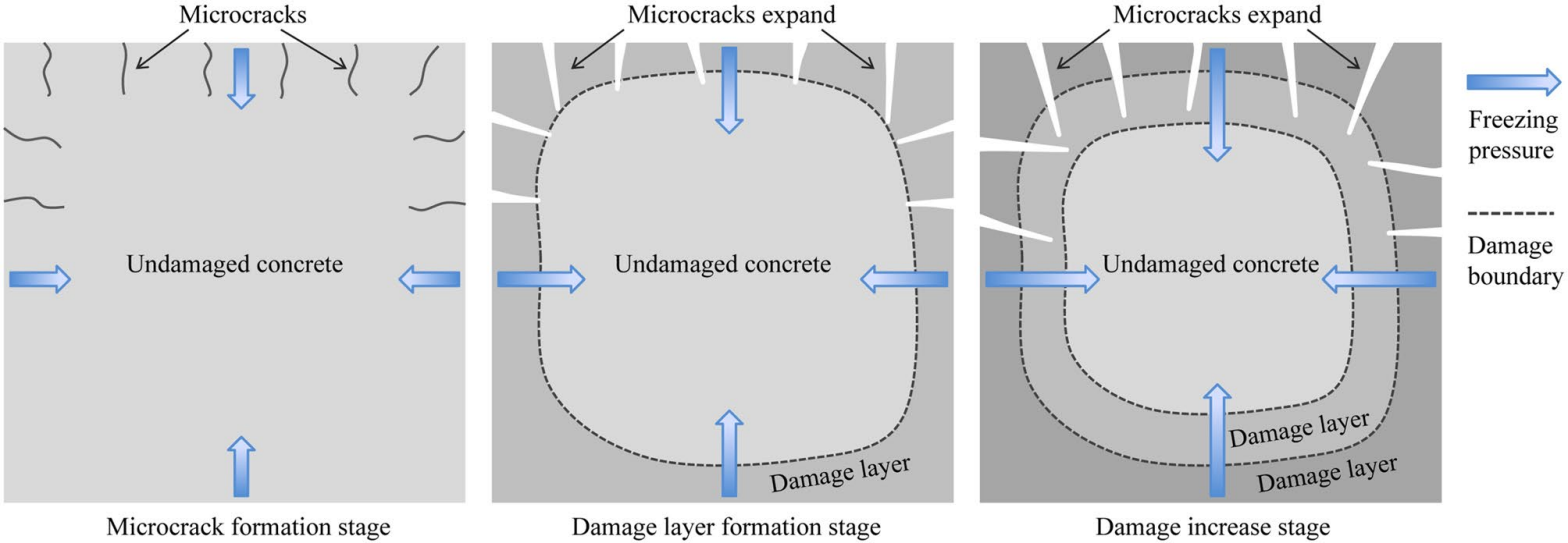
The process of damage evolution in SFRRC.

Figure 10 illustrates the process of damage evolution in SFRRC. During repeated F-T cycles, the outer layer of SFRRC is initially affected by freezing pressure, which leads to the formation of microcracks once the pressure exceeds the tensile strength of the concrete. As the number of F-T cycles increases, these microcracks gradually expand, causing a progressive deterioration in the internal microstructure of the concrete and ultimately resulting in the formation of a damaged layer. The progression and accumulation of this damage advance the boundary of the damage layer into the interior of the concrete, which is reflected by an increase in *H*_f_. This degradation can be observed macroscopically as a reduction in strength and *V*_f_.

**Figure 11:**
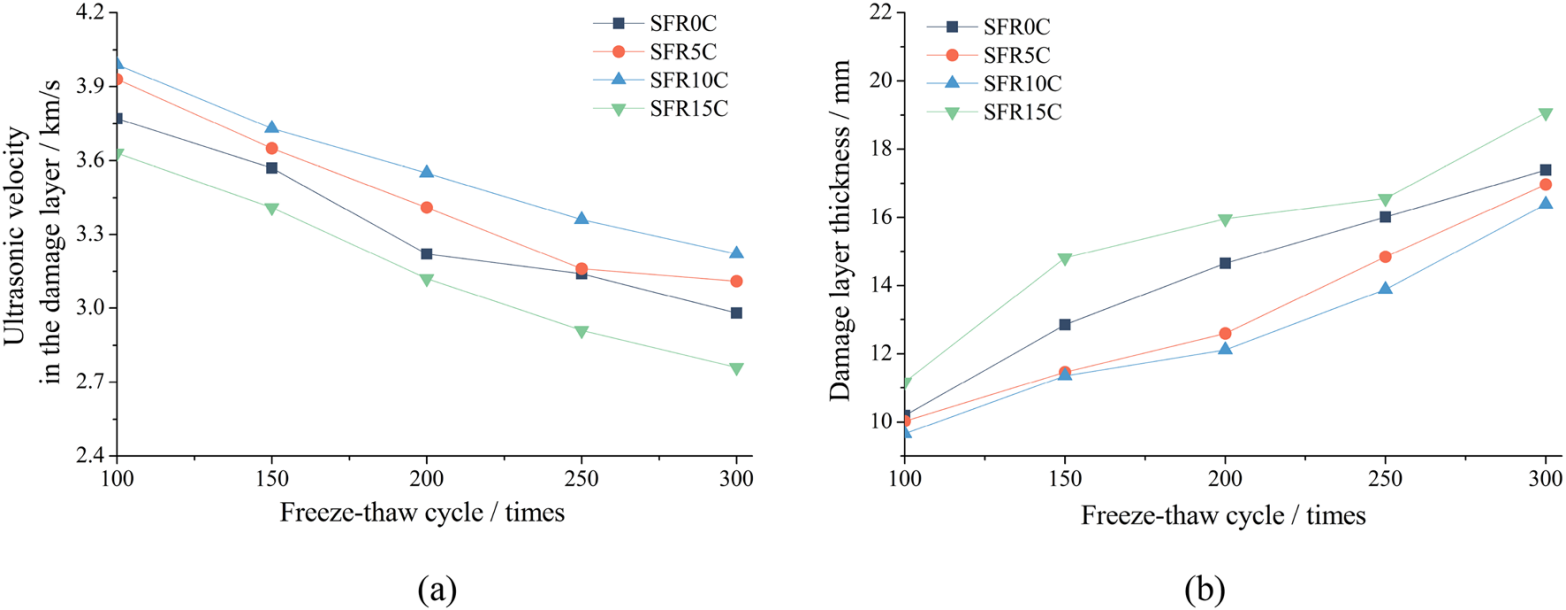
Change in damage layer thickness of SFRRC: (**a**) The *V*_f_; (**b**) The *H*_f_.

The variations of ultrasonic velocity and *H*_f_ in SFRRC with different rubber contents subjected to F-T cycles are illustrated in Fig. 11. As the experiment progresses, there is a gradual increase in the *H*_f_, while the *V*_f_ gradually decreases. After undergoing 300 F-T cycles, measurements reveal that the respective *H*_f_ values for SFR0C, SFR5C, SFR10C and SFR15C are recorded as 17.39 mm, 16.97 mm, 16.39 mm and 19.07 mm. It can be observed that SFR10C demonstrates superior frost resistance performance due to its smallest *H*_f_ and minimal reduction in the *V*_f_. However, once the rubber content reaches 15%, there is a noticeable increase in *H*_f_, which exceeds that observed in SFR0C without rubber. The change in the *H*_f_ resulting from F-T cycles corresponds to the experimental results illustrated in Figs. 7, 8, 9, as evident from the data. This indicates that a higher rubber content negatively impact the frost resistance capability of SFRRC. Due to the presence of a large number of microcracks and pores in the damaged layer, as these defects accumulate and expand, the originally solid concrete in the affected areas transforms into gas phase spaces^32^. This results in a decrease in *V*_f_, as ultrasound propagates more slowly in air compared to concrete. Similar findings have been reported in reference^36^, which observed that under sulfate attack and F-T conditions, the *H*_f_ of recycled concrete increases while *V*_f_ gradually decreases with an increasing number of F-T cycles.

**Figure 12:**
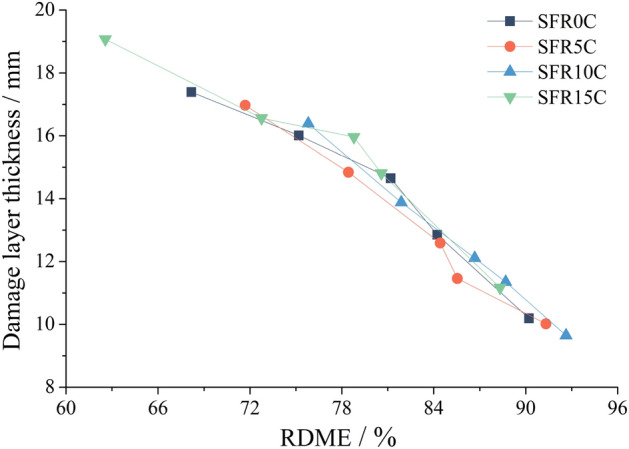
Relationship between *H*_f_ and RDME.

The experiment findings indicate that the fluctuation pattern of *H*_f_ aligns with the accelerated decrease stage of RDME, as shown in Fig. 8. This suggests a significant increase in the damage level of SFRRC after 100 F-T cycles. Both *H*_f_ and RDME are measured using ultrasonic techniques, and Fig. 12 illustrates the correlation between RDME and *H*_f_ in SFRRC subjected to F-T cycles. The graph clearly demonstrates a gradual increase in *H*_f_ as RDME decreases, indicating a strong association between these two factors. During F-T cycles, progressive deterioration occurs in SFRRC from its surface towards its interior. Therefore, utilizing *H*_f_ as an evaluation parameter can effectively assess the extent of degradation in SFRRC. Previous studies^31,32^ have also indicated that recycled aggregate concrete or coal gangue concrete exposed to F-T conditions exhibit increased severity of damage and reduced compactness, which corresponds to an increase in *H*_f_.

### Calculation of the compressive strength in damage layer

As the experiment progresses, an increase in the *H*_f_ of SFRRC leads to the emergence of defects in its meso-structure, resulting in a gradual decrease in the strength of the damage layer. This directly affects the bearing performance of the SFRRC structure. The calculation results for the *f*_d_ of SFRRC are presented in Table 4. It is evident that the *f*_d_ gradually decreases with an increase in F-T cycles. With an increase in F-T cycles, there is a gradual reduction in the *f*_d_. After undergoing 300 cycles, the damage layer of SFR0C (without rubber content) exhibits a reduction in compressive strength to approximately 47.33%, while for SFR5C, SFR10C, and SFR15C, the decrease amounts to around 40.09%, 36.03%, and 53.56% respectively. In comparison to the test results depicted in Fig. 9, the reduction in *f*_d_ is more pronounced. SFR15C exhibits the highest rate of strength reduction in its damage layer, surpassing even that of SFR0C without rubber. The observation suggests that SFR15C undergoes significant deterioration, indicating that the damage layer is the weak area of SFRRC when exposed to F-T conditions. Therefore, effectively addressing deterioration in the damage layer is essential for improving the mechanical properties and long-term durability of SFRRC.

**Table 4:** Calculated results of the *f*_d_ of SFRRC.

Sample types	F-T cycles	*A*/mm^2^	*A*_c_/mm^2^	*A*_d_/mm^2^	$f_{{\text{c}}}^{\prime}$*/*Mpa	*f*_c_*/*Mpa	*f*_d_*/*Mpa
SFR0C	100	10,000	6339.34	3660.66	44.4	46.8	40.24
	150	10,000	5520.49	4479.51	41.9	46.9	35.74
	200	10,000	4998.49	5001.51	39.8	46.9	32.70
	250	10,000	4621.28	5378.72	37.1	47.0	28.59
	300	10,000	4253.65	5746.35	34.2	47.1	24.65
SFR5C	100	10,000	6393.60	3606.40	39.2	40.8	36.36
	150	10,000	5941.33	4058.67	37.6	40.9	32.77
	200	10,000	5598.03	4401.97	35.4	40.9	28.41
	250	10,000	4944.90	5055.10	33.8	41.1	26.66
	300	10,000	4363.92	5636.08	31.8	41.3	24.44
SFR10C	100	10,000	6512.49	3487.51	38.5	39.8	36.07
	150	10,000	5975.29	4024.71	36.9	40.1	32.15
	200	10,000	5742.61	4257.39	34.9	40.1	27.89
	250	10,000	5218.62	4781.38	33.8	40.3	26.71
	300	10,000	4518.53	5481.47	32.3	40.6	25.46
SFR15C	100	10,000	6031.08	3968.92	33.9	37.1	29.04
	150	10,000	4953.34	5046.66	32.5	37.3	27.79
	200	10,000	4634.89	5365.11	30.3	37.5	24.08
	250	10,000	4472.93	5527.07	26.9	37.6	18.24
	300	10,000	3826.66	6173.34	25.1	37.8	17.23

### Microscopic morphology of SFRRC

**Figure 13:**
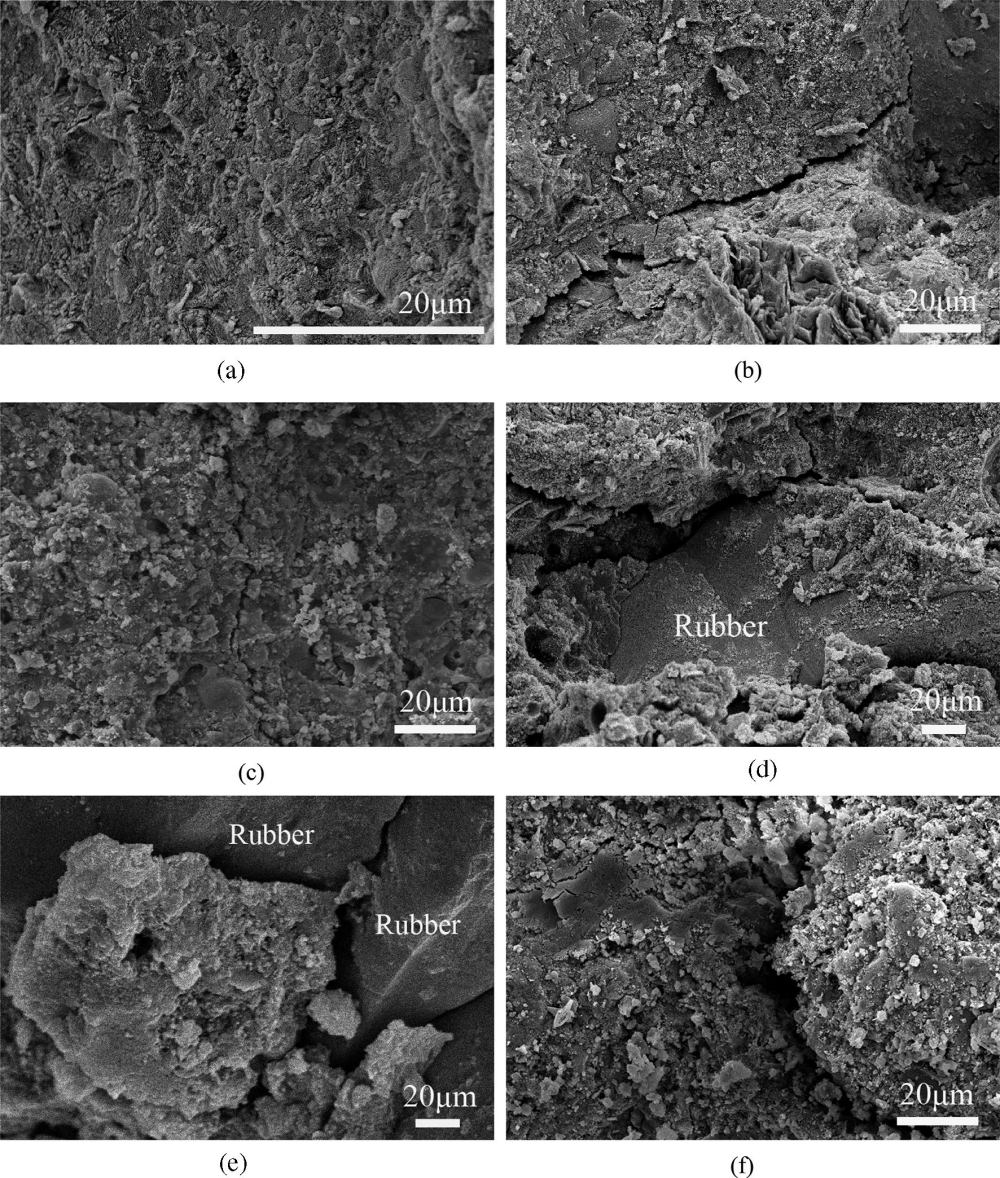
Microcosmic morphology of SFRRC specimens during F-T test: (**a**) Undamaged specimen; (**b**) Microcracks were observed in SFR0C after 200 cycles; (**c**) Microcracks were observed in SFR10C after 200 cycles; (**d**) ITZ between rubber and cement matrix; (**e**) ITZ between rubber and cement matrix; (**f**) Microcracks expand and the structure is obviously loose in SFR15C after 300 cycles.

SEM was conducted on the SFRRC samples both before and after undergoing freezing–thawing cycles to observe their microstructure, as depicted in Fig. 13. It is evident that the hydration products in SFRRC have cemented together to form a continuous phase prior to F-T damage. The overall structure appears uniform and compact without any microcracks, as depicted in Fig. 13a. During the F-T test process, the walls of SFRRC holes are subjected to both hydrostatic and osmotic pressures. In situations where the tensile stress exceeds the concrete's tensile strength, microcracks form within the SFRRC. Figure 13b and c respectively illustrate the presence of cracks in SFR0C and SFR10C after 200 cycles. The crack width of SFR10C is found to be smaller compared to that of SFR0C. As an elastic material, rubber particles exhibit excellent resistance to compressive deformation, thereby mitigating tensile stress caused by F-T cycles. Consequently, the progression of cracks in concrete is effectively hindered.

The frost resistance properties of concrete are further improved by the air-entrainment effect caused by rubber particles, which is evident in the reduced mass loss, RDME loss, and compressive strength loss exhibited by SFRRC. Additionally, a decrease in *H*_f_ is also achieved. However, due to its hydrophobic nature, rubber does not participate in the hydration reaction of cement. The primary bonding mechanism between rubber particles and the cement matrix is physical adhesion rather than strong chemical bonding^37^. Consequently, the interfacial transition zone (ITZ) between them becomes a weak area, as depicted in Fig. 13d. The large amount of rubber increases the number of micropores and the ITZs within SFR15C, resulting in a noticeable decline in its bonding strength at the interface and frost resistance. The limited adhesion between rubber and cementitious materials in SFRRC makes it prone to crack formation within the ITZs, with a tendency for the cracks to widen further (Fig. 13e). After undergoing 300 cycles, the SFR15C experiences an expansion and increase in internal cracks. Moreover, the cracks within the pores interconnect, resulting in a loosening of the structure of hydration products as depicted in Fig. 13f. Consistent with the macroscopic performance test results mentioned above, the SFRRC with a 15% rubber content exhibits significant deterioration. The macroscopic properties of SFRRC are inferred to be influenced by its microscopic structure.

### Analysis of the pore structure

**Figure 14:**
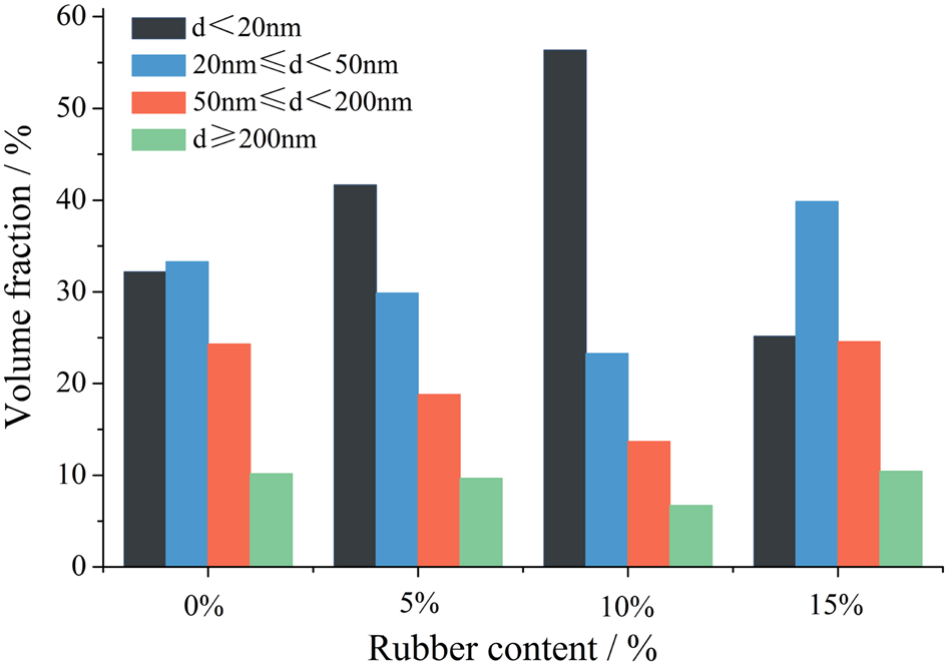
The influence of varying rubber content on the distribution of pore size.

Table 5 presents the parameters of SFRRC's pore structure obtained through mercury intrusion testing, while Fig. 14 illustrates the distribution of pore sizes. The incorporation of rubber particles is observed to increase the number of enclosed and fine bubbles in SFRRC, thereby enhancing its porosity and effectively functioning as an air-entraining agent. However, the macro performance of SFRRC is demonstrated by a reduction in compressive strength. The SFR0C without rubber exhibits minimal porosity, but it has a relatively large average pore size. Based on the data presented in Fig. 14, it is evident that SFR0C exhibits a reduced number of harmless (d < 20 nm) and less harmful (20 nm ≤ d < 50 nm) pores compared to SFR5C and SFR10C. Conversely, there is a higher proportion of harmful (50 nm ≤ d < 200 nm) and more harmful (d ≥ 200 nm) pores in SFR0C. This is reflected in the decreased frost resistance of SFR0C in terms of its macro performance.

**Table 5:** Pore structure parameters of SFRRC with different rubber contents.

Sample types	Total porosity/%	Total pore volume/(ml/g)	Total pore area/(m^2^/g)	Critical pore diameter /nm	Average pore diameter /nm	Most probable pore diameter /nm
SFR0C	11.21	0.0565	15.61	27.63	43.51	48.27
SFR5C	16.55	0.0817	22.75	33.17	35.37	38.81
SFR10C	13.39	0.0661	17.56	25.56	26.78	30.35
SFR15C	18.43	0.0897	26.93	41.87	47.86	54.13

Compared to SFR5C, the number of harmless pores in SFR10C increases by 35.23%, while the numbers of harmful and more harmful pores decrease by 27.20% and 30.71% respectively. Due to the hydrophobicity of rubber particles, an air–water film forms on the contact surface with mortar during the mixing process. The large pores on the surface of rubber particles are uniformly dispersed into stable and closed micro-pores under water adsorption^38^. Meanwhile, the air–water film attached to the rubber particles also participates in the hydration reaction of cementitious materials, further refining the pore structure^39^. Therefore, the data indicates that SFR10C demonstrates a decrease in total porosity, total pore volume, and area compared to SFR5C. The literature^24^ also notes that as the rubber powder content increases from 5 to 10%, the total porosity of SFRRC decreases to a certain extent, and the rate of increase in total porosity diminishes with an increasing number of F-T cycles. Furthermore, the increase in the ratio of harmless and less harmful pores also contributes to improving the frost resistance capability in SFR10C. The most favorable pore structure and the optimal frost resistance performance of SFRRC are achieved with a rubber content of 10%.

However, when the rubber content is increased to 15%, the number of harmless pores with d < 20 nm in SFR15C decreases by 53.20% compared to SFR10C, while the proportions of pores with sizes ranging from 20 nm ≤ d < 50 nm, 50 nm ≤ d < 200 nm, and d ≥ 200 nm increase notably. Therefore, a significant increase in pore structure parameters of SFR15C is observed. Specifically, compared to SFR10C, there is a 37.64% increase in total porosity, a 35.70% increase in total pore volume, and a 53.36% increase in total pore area. Moreover, both the average and most probable pore diameters in SFR15C demonstrate a significant increase, accompanied by an observed rise in the proportion of harmful and more harmful pores. In terms of macroscopic performance, this results in a significant decrease in both RDME and compressive strength for SFR15C, as well as an increase in *H*_f_. It is evident that the addition of rubber in an appropriate volume ratio improves the pore structure of SFRRC. However, excessive rubber content adversely affects both the pore structure and frost resistance of SFRRC.

## Conclusions

1. During the F-T cycles, SFRRC experiences a slow increase followed by a rapid increase in mass loss rate. While, the RDME and compressive strength exhibit a slow decrease followed by a rapid decrease. As the experiment progresses, there is a gradual reduction in ultrasonic velocity within the damage layer of SFRRC, accompanied by an increase in *H*_f_ and significant decline in compressive strength of the damage layer.
2. The frost resistance of SFRRC in water gradually improves as the rubber content increases from 0 to 10%, with SFR10C exhibiting the highest level. However, when the rubber content is increased to 15%, there is a noticeable decrease in frost resistance for SFR15C compared to SFR0C without the inclusion of rubber.
3. The formation of microcracks in SFRRC is attributed to the development of hydrostatic pressure and osmotic pressure during the experiment. As the experiment progresses, the cracks gradually expand and increase, ultimately leading to a loose structure of concrete hydration products. Rubber effectively mitigates the propagation of microcracks resulting from internal frost heave stress within SFRRC, thereby enhancing its frost resistance capabilities.
4. The incorporation of an appropriate amount of rubber can optimize the pore structure of SFRRC by increasing the proportion of harmless and less harmful pores, as well as reducing the average pore size. This enhancement subsequently improves the durability of SFRRC in F-T environments. However, a high rubber content (15%) in results in a significantly worse pore structure and decreased frost resistance performance.

In this paper, we only report on the impact of different rubber contents on the durability degradation of SFRRC under F-T cycles in water. Future research could further investigate the influence of various rubber particle sizes or steel fiber contents on the frost resistance of SFRRC. Additionally, it is also worth considering the effects of other common fiber types or hybrid fibers on the frost resistance of rubber concrete.

## Data Availability

All data generated or analyzed during this study are included in this published article.
